# Spectral Sensitivity Measured with Electroretinogram Using a Constant Response Method

**DOI:** 10.1371/journal.pone.0147318

**Published:** 2016-01-22

**Authors:** Fernando Allan de Farias Rocha, Bruno Duarte Gomes, Luiz Carlos de Lima Silveira, Sonia Limara Martins, Renata Genaro Aguiar, John Manuel de Souza, Dora Fix Ventura

**Affiliations:** 1 Instituto de Ciências Biológicas, Universidade Federal do Pará, Belém, Pará, Brazil; 2 Instituto de Psicologia, Universidade de São Paulo, São Paulo, São Paulo, Brazil; 3 Núcleo de Medicina Tropical, Universidade Federal do Pará, Belém, Pará, Brazil; 4 Universidade Ceuma, São Luís, Maranhão, Brazil; University of Sussex, UNITED KINGDOM

## Abstract

A new method is presented to determine the retinal spectral sensitivity function *S*(*λ*) using the electroretinogram (ERG). *S*(*λ*)s were assessed in three different species of myomorph rodents, Gerbils (*Meriones unguiculatus*), Wistar rats (*Ratus norvegicus)*, and mice (*Mus musculus*). The method, called AC Constant Method, is based on a computerized automatic feedback system that adjusts light intensity to maintain a constant-response amplitude to a flickering stimulus throughout the spectrum, as it is scanned from 300 to 700 nm, and back. The results are presented as the reciprocal of the intensity at each wavelength required to maintain a constant peak to peak response amplitude. The resulting *S*(*λ*) had two peaks in all three rodent species, corresponding to ultraviolet and M cones, respectively: 359 nm and 511 nm for mice, 362 nm and 493 nm for gerbils, and 362 nm and 502 nm for rats. Results for mouse and gerbil were similar to literature reports of *S*(*λ*) functions obtained with other methods, confirming that the ERG associated to the AC Constant-Response Method was effective to obtain reliable *S*(*λ*) functions. In addition, due to its fast data collection time, the AC Constant Response Method has the advantage of keeping the eye in a constant light adapted state.

## Introduction

There are several studies in vision that require knowledge of the wavelength range within which the retina is able to function. In fact, determining this range by spectral sensitivity functions *S*(*λ*) for a given species is the core of any experiment that utilizes visual stimulation to obtain either behavioral or physiological parameters. The *S*(*λ*) gives information that might suggest different types of retinal processing as well as clues about behavioral priorities when the animal is in its natural environment. Classically, one can determine the photoreceptor *S*(*λ*) directly from measurements performed in outer segment of the photoreceptor cell through microspectrophotometry [1–10] or indirectly from behavioral methods [11–18]. There are also a number of electrophysiological methods to determine photoreceptor spectral sensitivity (reviewed in [19]). De Souza *et al*.[20], developed an efficient apparatus to make measurements of *S*(*λ*) functions from intracellular recordings of retinal cells called AC Constant Response Method (AC method). This was a modification of previous procedures used in photoreceptor measurements of *S*(*λ*), that relied on the cell’s steady response to light (DC methods) [21].

The DC constant-response method adjusts the intensity of a steady light to keep the response amplitude constant as wavelength is swept from one end to the other end of the spectrum. This method is applicable to photoreceptor cell responses, whose amplitude remain constant to a light stimulus of constant intensity. In the DC constant response method a computer steps the spectrum from ultraviolet to red or vice-versa. After each wavelength change, the response may increase or decrease in amplitude. The computer is programmed to step a neutral density wedge up or down to correct for this amplitude change, until the photoreceptor response crosses a constant criterion value [19, 21].

De Souza *et al*.[20] were interested in being able to determine *S*(*λ*) of other cell types, such as bipolar cells, whose response to light decreases over time, rather than being a step change, as in the photoreceptor. They thus devised a method in which an intermittent stimulus replaces the steady light which is presented in the DC method. When using the AC method the cell is stimulated with a flickering light, the computer then measures the peak-to-peak amplitude of the cell response and adjusts the intensity of the light to keep this response amplitude matched to a predetermined criterion. As in the DC method, this is effected by automatically controlling the position of a neural density filter to decrease or increase light intensity.

Ventura *et al*. [22,23] used the AC method to indirectly estimate *S*(*λ*) of UV cones in the turtle (*Trachemys scripta elegans*) by recording it from horizontal cells and comparing different chromatic adaptations. The UV function thus obtained had its maximum sensitivity at 372 nm—a result exactly confirmed by concomitant investigation using microspetrophotometry (MSP) [6]. The coincidence of *S*(*λ*) peaks obtained with MSP and electrophysiology ratified the reliability of the AC method as a valuable tool to measure spectral sensitivity functions.

The ERG has also been extensively used for measurements of *S*(*λ*) in a variety of species [11–15,17,18,24–32]. One important approach using ERGs was the determination of *S*(*λ*) curves by flicker photometry developed by Jacobs *et al*. [33]. This was the color substitution method in which light from two beams, a flickering test and a reference beam, are interleaved and the intensity of the test beam is varied until the responses to the two beams are equated. To determine *S*(*λ*) the procedure is repeated for different wavelengths in the test beam.

In the present study, we used a modified version of the AC Constant Response Method to measure spectral sensitivity from mouse, rat, and gerbil using ERG. *S*(*λ)* measured with ERG contains pooled contributions of different photoreceptor types summed with the contribution of other cells in the retinal network, rather than the output of single photoreceptors or horizontal cells as in the previous studies that used this method to measure *S*(*λ*) through intracellular recordings in the eyes of bees and turtles [19,20,22,23]. The main advantages of using ERG as information source are that it is a simple procedure to implement in comparison with intracellular recordings and that it is a non invasive method. As such, it allows easy adaptation to other mammalian species.

## Materials and Methods

### Subjects

Experiments were performed on three rodent species: albino Wistar rat (*Rattus norvegicus*), gerbil (*Meriones unguiculatus*), and mouse (*Mus musculus*). The animals, all adults at the time of testing (about 3 months old), were housed in cages measuring 41 x 34 x 16 cm, two animals per cage, food and water ad libitum, and kept on a 12h light / 12h dark cycle with ambient light. Animal handling and care complied with the Society for Neuroscience guidelines, also recommended by the Brazilian Society for Neuroscience and Behavior. The procedure was approved by the Ethic Commission for Research with Animals, Psychology Institute, University of São Paulo, protocol #07/56844/-1, 19^th^ March 2008.

The animals were anesthetized with an intramuscular injection of a mixture of xylazine hydrochloride (21 mg/kg) and ketamine hydrochloride (108 mg/kg). The pupil was dilated with atropine sulfate (0.04%) eye drops (about 30 min before start of measurements). The animals were positioned in a head restraint apparatus and aligned with the optical system. ERGs were recorded with DTL electrodes (Diagnosys LLC, Lowell, MA, USA) placed over the corneal surface after applying drops of 1% methylcellulose. Ground (Grass E5 disc electrode; Grass-Telefactor, West Warwick, RI, USA) and reference electrodes (Grass-Telefactor) were placed on the forehead and external canthi, respectively. Retinal electrical potentials were amplified (Grass-Telefactor) with a band-pass set between 0.3–100 Hz, monitored on an oscilloscope (TDS 210; Tektronix, Richardson, TX, USA), and continuously digitized at a rate of 1 kHz by a computer equipped with a data-acquisition board (National Instruments, Austin, TX, USA). Before cone ERGs were recorded, the animals were light adapted for 10 min to assure maximal cone output and moreover the recordings were made in a room illuminated by ceiling-mounted fluorescent lamps (150 lx). This procedure was used in previous studies that aimed to have measured cone responses without rod influence [17,33].

### Apparatus

The determination of *S*(*λ*) was made using the equipment and general procedures described earlier [22,34]. In brief, an intermittent stimulus was delivered from an optical system, the output of which was presented in Maxwellian view (circular field 57° in diameter). A beam of monochromatic light originating from a monochromator (38-86-79; Bausch & Lomb, Tampa, FL, USA) equipped with a 75-W xenon arc lamp was used. A circular 4 log-unit neutral-density wedge was used to adjust light intensity (maximum intensity about 4.6 x 10^16^ quanta/s/cm^2^). In the pupil plane, the optical system spectral output was calibrated with a radiometer (IL 1700 with modelED033 photodetector; International Light Technologies, Peabody, MA, USA) at all combinations of wavelength and position of the neutral-density wedge.

### Software

The Spectral Analysis software, written in Visual Basic programming language (Microsoft, Redmond, WA, USA), had a number of windows that allowed the experimenter to set up and run spectral scans. The software controlled the flicker frequency via either a shutter or current modulation of the xenon lamp. It controlled the monochromator, advancing it in regular nm steps (usually 4 nm, but the step size could be set by the experimenter). A preset value of criterion amplitude was used by a comparator in the software to send a command to drive the neutral density wedge up or down, every time there was an amplitude change, resulting from a wavelength change. A spectral scan consisted of a run from 300 to 700 nm, i.e., there were 100 measurements of the wedge position necessary to keep constant the peak to peak amplitude of the response to the flickering light. Results of the spectral scans were plotted on-line as *S*(*λ*) plots and were automatically saved to disk. *S*(*λ*) curves could also be averaged and saved. In order to calculate *S*(*λ*) each of the 100 wedge positions determined in a spectral run was translated into a quantum flux value. These values were found in a calibration table of relative quantum flux determined for each monochromator-wedge combination, i.e., 101 monochromator positions times 256 wedge positions = 25856 values. Relative quanta were determined with a separate program that stepped the monochromator and wedge to each of the 25,856 combinations, The radiance input at the position of the eye was measured with an International Light IL700 radiometer with a PMD271D photomultiplier detector and converted to quantum fluxes.

### Functional Description

The preparation was kept in a Faraday cage. We initially ran a spectral series consisting of monochromatic light flashes (at -1.5 log below the maximum intensity) from 340 to 560nm with intervals of 20 nm between flashes and then we proceeded to the determination of the *S*(*λ*) with the AC Constant Response method [19,20]. In this method, a flickering light was produced by a shutter (100% modulation). The resulting ERG was periodic and varied in amplitude across the light spectrum. The system measured the peak-to-peak response voltage (PPV) at a given wavelength and compared it to a pre-set criterion value. At each wavelength the periodic response to the flickering stimulus was digitized at 1000 samples/s and smoothed by averaging 16 consecutive samples; the peak-to-peak amplitudes of the smoothed samples were found and compared to the previously set criterion. If they were unequal, the wedge was stepped to a denser or a less dense position depending on the sign of the difference. After the criterion value had been crossed the wedge position corresponding to the closest match was recorded. The system could be programmed to average two or more consecutive peak-to-peak measurements before comparison with the criterion. Wavelengths were sampled from 300 to 700 nm, or vice-versa, in 4 or 12 nm steps.

Each *S*(*λ*) curve is an average of 10–15 spectral scans per animal. The frequency of stimulation was adjusted in the range of 4–12 Hz, the criteria of ERG response amplitude was set to 4μV amplitude.

### Spectral Sensitivity Curves Estimate

We used the residual method to determine the sensitivity curves and peak maximum sensitivities with the support of software PeakFit v. 4.12 (SeaSolve Software, Bangalore, Karnataka, India). This method is highly sensitive for the detection of peaks and is often used to find peak sensitivity in spectrographic or chromatographic among others applications [35–38].

**Fig 1:**
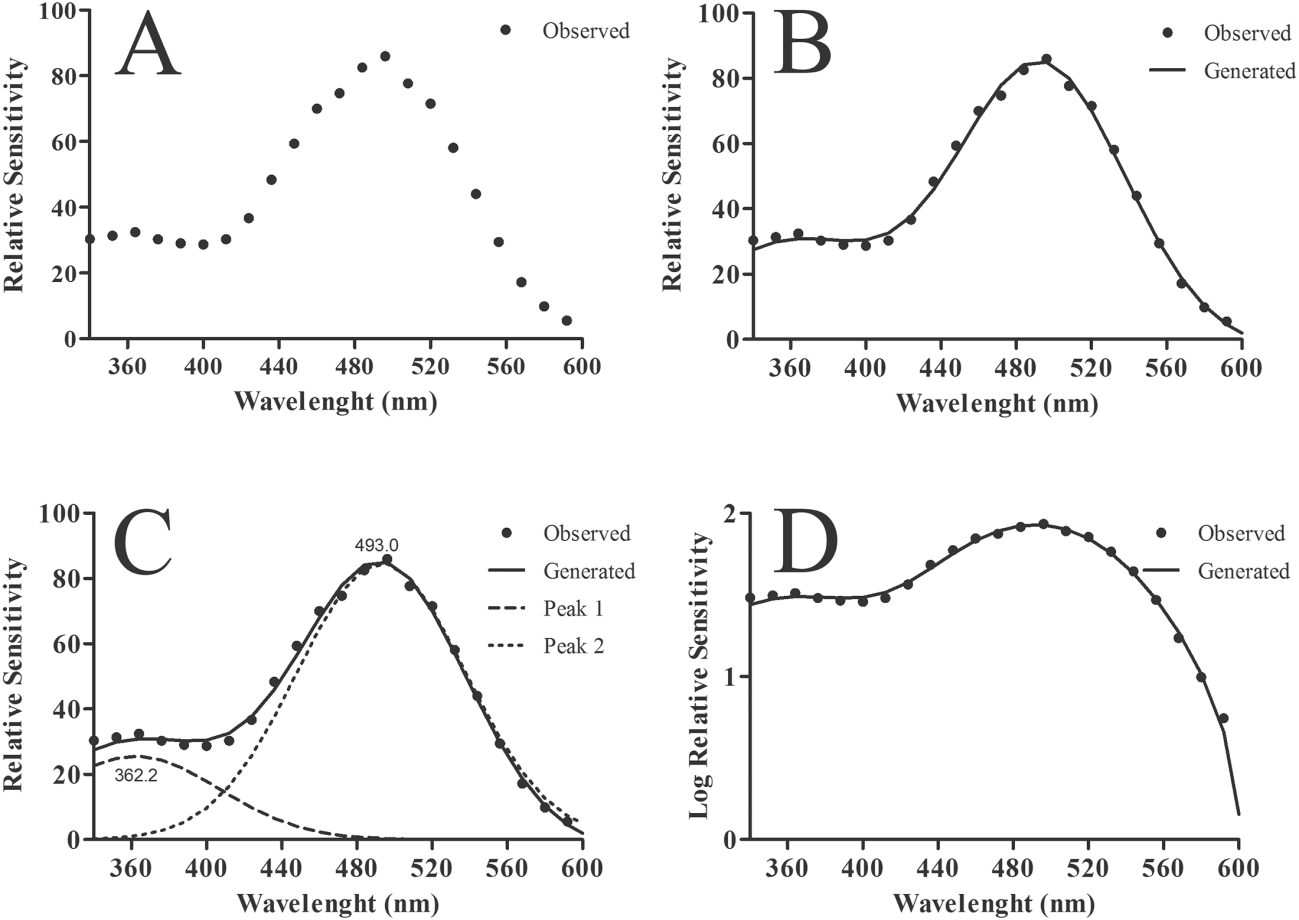
Sensitivity curves determined by the residual method. **(A)** Data obtained by using the AC Constant-Response Method for the gerbil. **(B)** Spectral sensitivity curve obtained using a Fast Fourier Transform (FFT) filter fitted to data points showed in (A). **(C)** Two peaks were found by fitting Gaussian normal curves to the FFT results. For the gerbil, the two peaks were located at 362 nm and 493 nm. **(D)** The same as in (B) showed in log scale.

Briefly, the residual procedure provided a curve fitting by using a Fast Fourier Transform (FFT) filter from the data obtained by the AC Constant-Response Method (Fig 1A and 1B). The peaks were placed at local maxima in a smoothed data stream (Fig 1C) using Gaussian curves. Fig 1 illustrates data obtained from gerbils, where two sensitivity peaks (*λ*_max_) were found: one in the UV range with a *λ*_max_ at 362 nm and another in the green region of the light spectrum with a *λ*_max_ at 493 nm.

## Results

**Fig 2:**
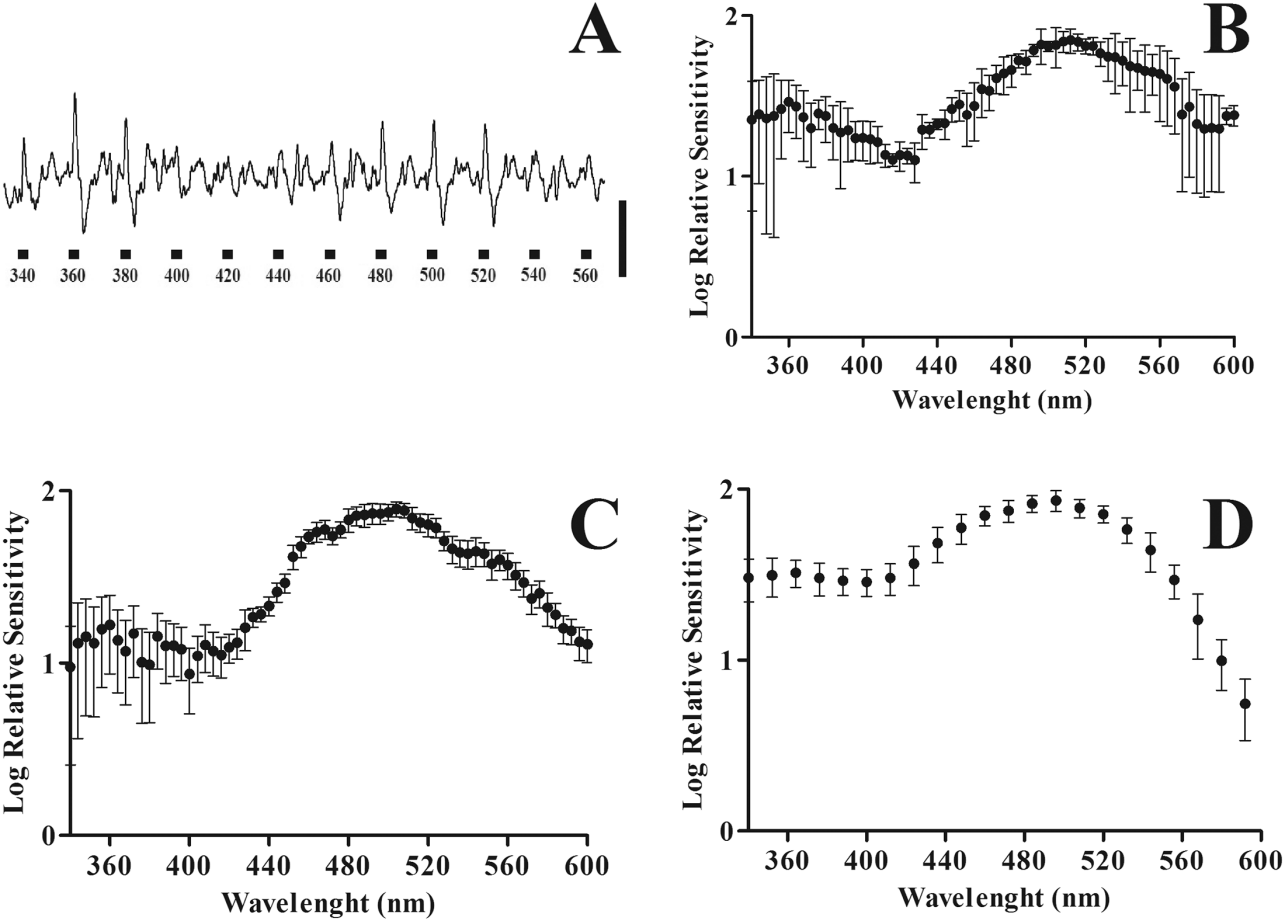
Spectral series and spectral sensitivity obtained by using the AC Constant-Response Method. **(A)** ERG responses obtained from a light-adapted mouse. ERG responses were driven by flashes of monochromatic equal quanta lights of different wavelength. Note responses to wavelengths in the UV and green ranges. **(B)** Mean spectral sensitivity for mice. Filled circles and bars represent means and standard deviations for n = 3 animals. **(C)** and **(D)** are the mean spectral sensitivities obtained for rats (n = 3) and gerbils (n = 3), respectively. Spectral sensitivity curves for mice and rats were obtained at 4 nm intervals while curves for gerbils were obtained at 12 nm intervals.

Fig 2A shows a spectral series of ERGs from a light adapted mouse eye, obtained with flashes from 340 to 560 nm of equal quanta (-2 log attenuation of the maximum intensity). Responses were larger between 360–380 nm. They became virtually absent between 400–420 nm and increased again in the range of 440–560 nm where the largest amplitude was found at 500 nm. This result was expected since according to the literature [27,29], mice have greater sensitivity in the UV and green ranges of the spectrum. An analogous pattern was observed for the rat and gerbil (data not shown).

### Spectral Sensitivities for Different Species of Rodents

The next step was to perform measurements of spectral sensitivity with the AC Constant-Response Method on different rodent species. In all procedures, three animals of each species were used and 10–15 scans were performed in each animal with wavelengths presented from 340 to 600 nm in 4 or 12 nm steps. Average curves representing the *S*(*λ*) function were then fitted to the data points.

Fig 2B shows the *S*(*λ*) functions obtained from mice. Two peaks were observed: a small one at 350–370 nm and a second large one between 500 and 520 nm. Peak values differed in sensitivity by approximately 0.5 log unit. For values larger than 520 nm, the sensitivity dropped sharply towards 600 nm. We did not observe significant responses for flashes above 620 nm.

The *S*(*λ*) functions measured in rats (Fig 2C) also showed two peaks. The maximum sensitivity occurred in the green range of the light spectrum. When compared with the curve obtained for mice, the sensitivity range had an offset of -10 nm, with the peak situated in the range of 490–510 nm. Another peak could be seen in the UV range. The peaks differed in sensitivity by 0.8 log. The spectral sensitivity curve measured in gerbil (Fig 2D) was similar to those found in mice and rats despite the fact that the flicker frequency used had been 12 Hz. Similarly, to results obtained in the other two rodents, two peaks were observed, one in the range of 480–500 nm and another in the UV range. The peaks differed in sensitivity for approximately 0.5 log.

**Fig 3:**
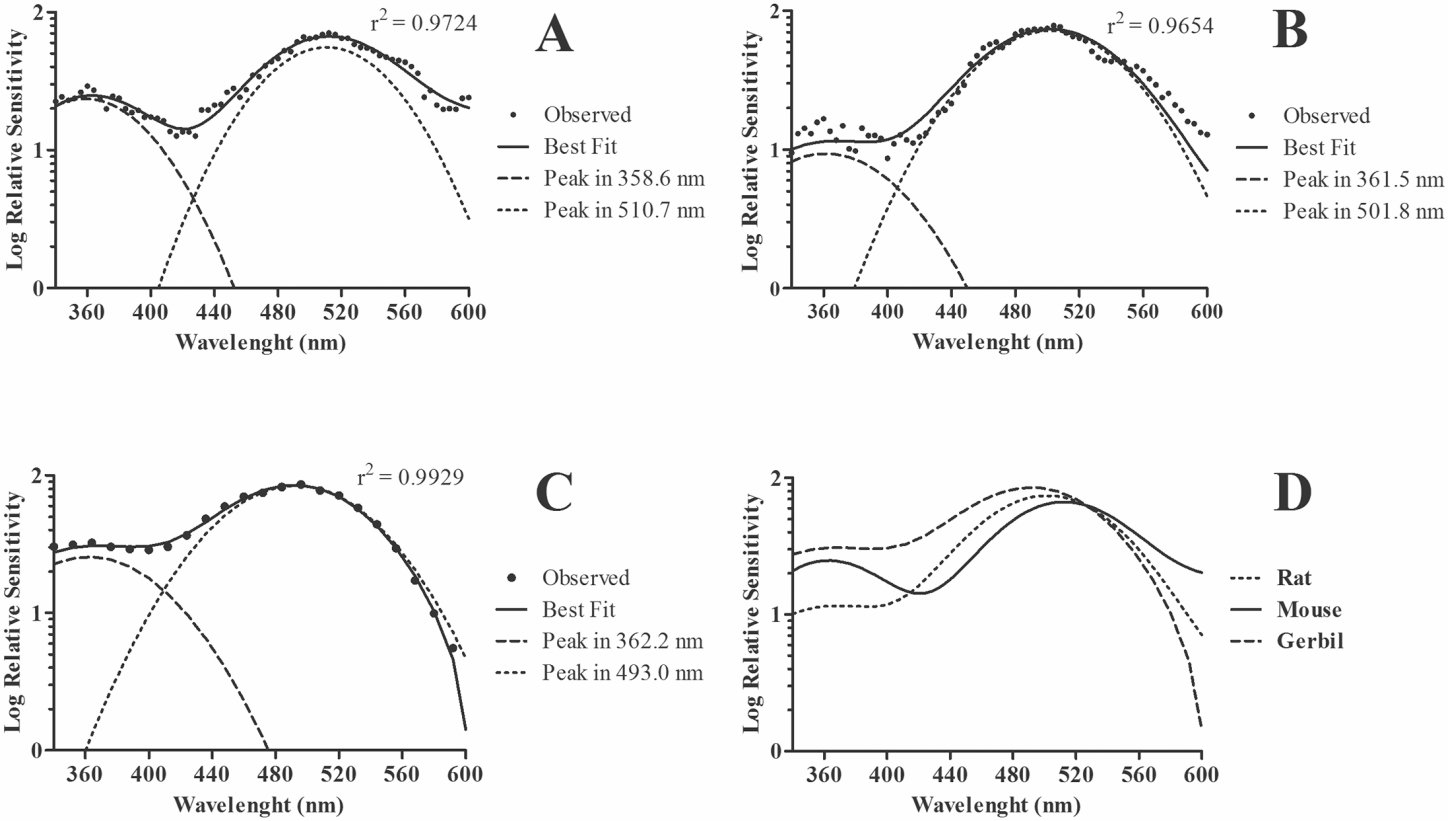
Mean spectral sensitivity curves for different species measured with the AC Constant-Response Method. **(A)** Mean *S*(*λ*) curves for mice with *λ*_max_ at 359 and 511 nm (n = 3). **(B)** Mean spectral sensitivity curves for rats with *λ*_max_ at 362 and 502 nm (n = 3). **(C)** Mean spectral sensitivity curves for gerbil with *λ*_max_ at 362 and 493 nm (n = 3). **(D)** Best fittings obtained for each species. For each species, spectral sensitivity curves directly obtained from FFT fits and underlying Gaussian curves representing individual UV and M cone spectral sensitivities are shown.

**Fig 4:**
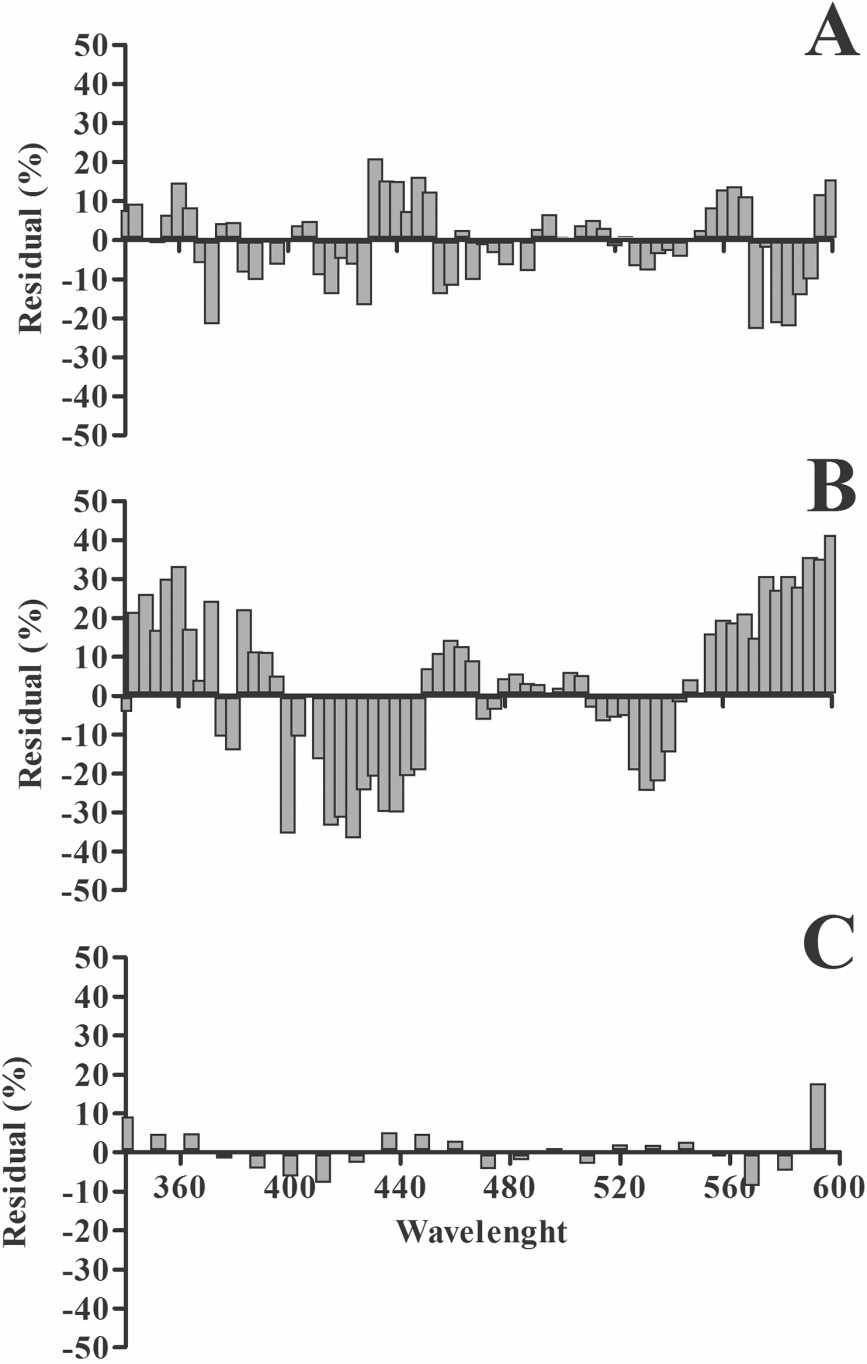
Residual analysis for the spectral sensitivity curves of different species. **(A)** and **(B)** Results for mice and rats, respectively, where large differences relative to the adjustment curve were observed. **(C)** Results for gerbils where the differences were small.

Once we had analyzed all data points for the discrete wavelength range we then applied the residual method to estimate fitting curves for *S*(*λ*) of each rodent species (see Material and Methods). Fig 3A shows *S*(*λ*) as a continuous curve for mice (*r*^*2*^ = 0.9724) and the dashed lines are Gaussian curves generated by curve adjustment. The maximum sensitivities were 359 nm and 511 nm. For rats, the *λ*_max_ was found at 362 nm and 502 nm (*r*^*2*^ = 0.9654). For the gerbil the best curve yielded *λ*_max_ at 362 and 492 nm (*r*^*2*^ = 0.9929). Fig 4 shows a residual plot for each species. The highest and lowest deviation between the fitted curve and the data was observed for gerbil and rats, respectively (Fig 4B and 4C).

## Discussion

The AC Constant-Response Method has been used in previous studies to estimate the spectral sensitivity of bees and turtles through intracellular recording of photoreceptors (bees) or horizontal cells (turtles) [20,22]. In bees, de Souza *et al*. [20] estimated *S*(*λ*) curves of three types corresponding to three photoreceptors (UV, blue, and green) peaking at 354, 430, and 518 nm. Another set of experiments that confirmed the reliability of the AC Constant-Response Method was performed by Ventura *et al*. [22], who described the spectral sensitivity for UV cones in turtles using intracellular recordings of horizontal cells. They found peak sensitivity at 372 nm for the UV cones. Simultaneously, Loew and Govardovskii found similar results using microspectrophotometry [6]. In the studies with turtles, the experiments were done with isolated eyes, using an eyecup preparation and required procedures to allow an adequate tissue survival. In the present study we extended the use of the AC Constant-Response Method for measurements using ERG which is a noninvasive measurement and thus enabled the calculation of *S*(*λ*) *in vivo*.

The ERG is a mass extracellular recording with a typical phasic response. The choice of the AC Constant-Response Method was adequate for this type of response because it is independent of the response type to be recorded, phasic or tonic [19,20]. Because the criterion in the AC method is a peak-to-peak amplitude rather than a voltage level [19,20], another advantage of the AC method is that it is insensitive to baseline changes during the recording session. These are common occurrences in electrophysiological recordings such as the ERG due to eye movements or other interferences. Moreover, ERGs elicited by a periodic stimulus (flicker ERG) are derived by both photoreceptors and bipolar cells when they are recorded at the cornea. Therefore, normal signaling of photoreceptors and bipolar cells as well as regular synaptic transmission from photoreceptors to bipolar cells are indispensable for the methods.

Nevertheless, the use of ERG associated with the AC Constant-Response Method showed high sensitivity, being able to detect spectral response of both cone populations (UV and M cones). These results showed that even in animals with low numbers of cones such as the rat, where cones correspond to about 1% of all photoreceptors [19], the method was still effective for photopic spectral sensitivity curve determination.

We were able to reproduce with the AC Constant-Response Method measurements of sensitivity in the UV range for the mouse and gerbil. Our results were very similar to those reported in previous publications by other authors. In our study, the fits for these animals showed peak sensitivity at 362 nm for the gerbil and 359 nm for the mouse. Peak sensitivity has been reported by Jacobs and collaborators to occur at 360 nm in gerbil [15] and 360 nm in mouse [29], in both cases they used the ERG flicker photometry method [33]. For the M cones we were able to measure the spectral sensitivity of the three species studied. Our results for the mouse and gerbil were very similar to those of other studies [15, 27, 29]. The peak sensitivity found for gerbils was 493 nm and this *λ*_max_ coincided with that found by Jacobs and Deegan [15]. For mice, the *λ*_max_ was 511nm and it is within the range of 509–512 nm described by Jacobs *et al*. [29].

Most studies that measured visual spectral sensitivity in albino mice have found curves shaped by M cone response [39–43]. Under light adaptation, ERG and behavioral results provided a *λ*_max_ near 500 nm [39,42]. Lewis and colleagues used fundus reflectometry to report *λ*_max_ at 505 nm [41]. In our study the *λ*_max_ at about 501.8 nm using the AC method is closer to the peaks reported by studies that used ERG. In addition, our method was able to show a second prominent elevation peaking at the UV range (362 nm). For comparison, the *λ*_max_ for pigmented rats in the UV range has been reported as being around 359 nm [17], which is only 3 nm apart from our peak measure.

Previous studies have reported peak differences for the middle wavelength part of the spectrum between pigmented and albino rats [39,40,43]. Similarly, in the middle wavelength range *λ*_max_ was higher (509 nm) for pigmented rats [17] compared to peak measured for albino rats in our study (501 nm). It confirms the accuracy of the AC method for spectral sensitivity measures. The results obtained for the albino rat are extremely important because of the wide use of these animals in physiological and behavioral studies of vision.

The residual analysis showed that the higher deviations from the fitted curves were found for rats followed by mice and by gerbil, with the lowest residual bounds. This discrepancy in the residual analysis could be attributed to the proportion of cones at each species relative to the total number of photoreceptors. The gerbil is a diurnal rodent whose photoreceptors comprise 13% of cones [44]. In contrast, in the retina of the nocturnal mice and rats the proportion of cones is only 1–3% [45,46]. The reduced number of cones in mice and rats compared to gerbil may have influenced the ERG amplitude adding a high cross-trials variability mainly in the UV range where the highest residual values were observed. The influence of the number of cones on the ERG variability found in our results finds support in other studies. Yang and cols [47] recently conducted a detailed study of scotopic and photopic ERG in gerbil and mouse and pointed out that under scotopic conditions, the ERG response was higher in the rat. However, in photopic conditions the amplitude of both the a-wave and b-wave were higher in the gerbil.

Since the animals used were dichromats, it is not possible to know from the recordings if the method can discriminate the peaks from primates M and L cones, including humans. However, we had previously performed chromatic adaptation experiments in intracellular recordings from horizontal cells in the turtle and by doing so we were able to separate the four photopigments that exist in that species. We were pleasantly surprised by the fact that the peak we found for the ultraviolet receptor coincided almost exactly with that measured by Loew and Gowardovskii using microspectrophotometry [7]. We are planning to extend the method to human recordings but this would require some adaptation of the optical system.

In the present study we have successfully used the AC Constant-Response Method to obtain spectral sensitivity curves from rodent species using the ERG. The reliability of the method has already been established in previous studies using intracellular measurements [19,20,22]. The extension of the method to include ERG measurements remarkably simplifies the procedure enabling the application to mammals, whose retinas have smaller cells and require an intensive care to be maintained alive when compared with the retina of reptiles or insects.

## Supporting Information

S1 ARRIVE Checklist ARRIVE Guidelines Checklist. (DOC)

S1 Dataset Rodent sensitivity curves obtained with the AC Constant-Response Method. (XLSX)
